# Hopital De La Pitié

**Published:** 1829-08

**Authors:** 


### HOPITAL DE LA PITIÉ.

### *Fistula of the Parotid Duct and Gland successfully treated.* By M. Beclard.

A youth, aged eighteen, lacerated his cheek by falling upon an iron stove. One of the upper screws penetrated the cheek, wounding the buccinator muscle near the masseter, though it does not appear that it reached the mouth. The wound healed rapidly, with the exception of a small fistula, through which there was a discharge of a clear liquid, especially while he was eating. This showed that the stenonian duct had been wounded. After having fruitlessly employed the several means which were directed by his attending surgeon, on the 21st of May, 1821, he placed himself under the charge of M. Beclard, in l'Hôpital de la Pitié. It had been three years since he had received the injury.

M. Beclard converted the fistula into a recent wound, by the excision of its sides; then, with a small trocar, he perforated the cheek from the wound, passing the instrument obliquely backwards. Having withdrawn the stilet through the canula, which remained in the perforation, he passed the end of a leaden wire. Having withdrawn the canula, he in the same manner made a second puncture; beginning, however, within the mouth, about three lines anterior to the former puncture, carrying the point of the instrument obliquely, till it passed into the wound near the place where the wire was inserted. The external end of the wire was passed through this opening into the mouth, being thus bent somewhat in the form of the letter V. The ends of the wire were twisted together, and the external wound was closed by a needle and the twisted suture. For the first three days, there was some distention and pain, owing to the saliva not passing at first very readily by the side of the leaden wire. On the fifth day, the saliva passed freely into the mouth, and the swelling was nearly gone; the external wound had united, and the needle was now removed. Still the internal fistula was not so complete as to carry off all the saliva while the patient was eating. The duct was distended into a kind of sac, producing, during his meals, a small tumor, which was, however, easily emptied into the mouth, by a very slight external pressure. The leaden wire remained inside the cheek, till it fell out in September, when the cure was complete.—*Archives Générales.*

